# Feasibility of Laparoscopically Assisted Percutaneous Endoscopic Gastrostomy in Patients for Whom Endoscopic Gastrostomy Is Difficult: A Retrospective Study

**DOI:** 10.7759/cureus.84193

**Published:** 2025-05-15

**Authors:** Akira Kameyama, Seijiro Yoshifuku, Masatake Miyamoto, Sasahara Kotaro, Otagiri Noriaki, Yasunori Nishida, Katsunori Tauchi

**Affiliations:** 1 Department of Gastrointestinal Surgery, Aizawa Hospital, Matsumoto, JPN

**Keywords:** gastrostomy, laparoscopically assisted peg, laparoscopy, open gastrostomy, percutaneous endoscopic gastrostomy

## Abstract

Background

Percutaneous endoscopic gastrostomy is a widely used technique for long-term enteral nutrition. However, some patients have anatomical or pathological conditions making standard percutaneous endoscopic gastrostomy infeasible. Traditionally, open gastrostomy (Open-G) has been performed in these cases, although it is more invasive.

Laparoscopically assisted percutaneous endoscopic gastrostomy (LAPEG) has recently been introduced as an alternative.

This study aimed to compare the surgical outcomes of LAPEG and Open-G in patients for whom percutaneous endoscopic gastrostomy was deemed difficult.

Methodology

A retrospective review was conducted of all patients undergoing gastrostomy at our institution between January 2016 and February 2025. Among 378 total gastrostomies, 16 were classified as *difficult*, meaning that endoscopic transillumination or the finger sign could not be confirmed, or preoperative imaging revealed organs overlapping the stomach.

Data collected included operative time, blood loss, and perioperative complications. Statistical analyses used chi-square and t-tests, with significance set at *P* < 0.05.

Results

Sixteen patients were analyzed (four LAPEG and 12 Open-G). Median operative times (49 minutes vs. 55.5 minutes) and blood loss (3.5 g vs. 5 g) did not differ significantly between the LAPEG and open groups, respectively.

Conclusions

Within the limitations of this small, retrospective study, LAPEG was comparable to Open-G in terms of operative time and surgical outcomes.

Future large-scale, prospective studies are necessary to validate these findings and further clarify the clinical benefits of LAPEG in patients with difficult indications for percutaneous endoscopic gastrostomy.

## Introduction

Gastrostomy plays a crucial role in patients who require long-term nutritional management but cannot maintain adequate oral intake. Percutaneous endoscopic gastrostomy is widely considered the standard approach due to its relatively low invasiveness and high success rate [1]. However, standard percutaneous endoscopic gastrostomy cannot be safely performed in certain patients with challenging anatomical or pathological conditions, such as extensive adhesions or an overlapping transverse colon. Traditionally, open gastrostomy (Open-G) has been performed for these *difficult* cases, but open surgery is more invasive and carries an increased risk of postoperative complications. Laparoscopically assisted percutaneous endoscopic gastrostomy (LAPEG) has emerged as an alternative, allowing adhesiolysis, direct visualization of the stomach, and precise placement of the gastrostomy tube under laparoscopic guidance. Although there is increasing interest in LAPEG, few studies have evaluated its clinical impact in *difficult percutaneous endoscopic gastrostomy* cases. Therefore, we conducted a retrospective analysis comparing Open-G and LAPEG in terms of operative time and complications [2].

## Materials and methods

**Figure 1:**
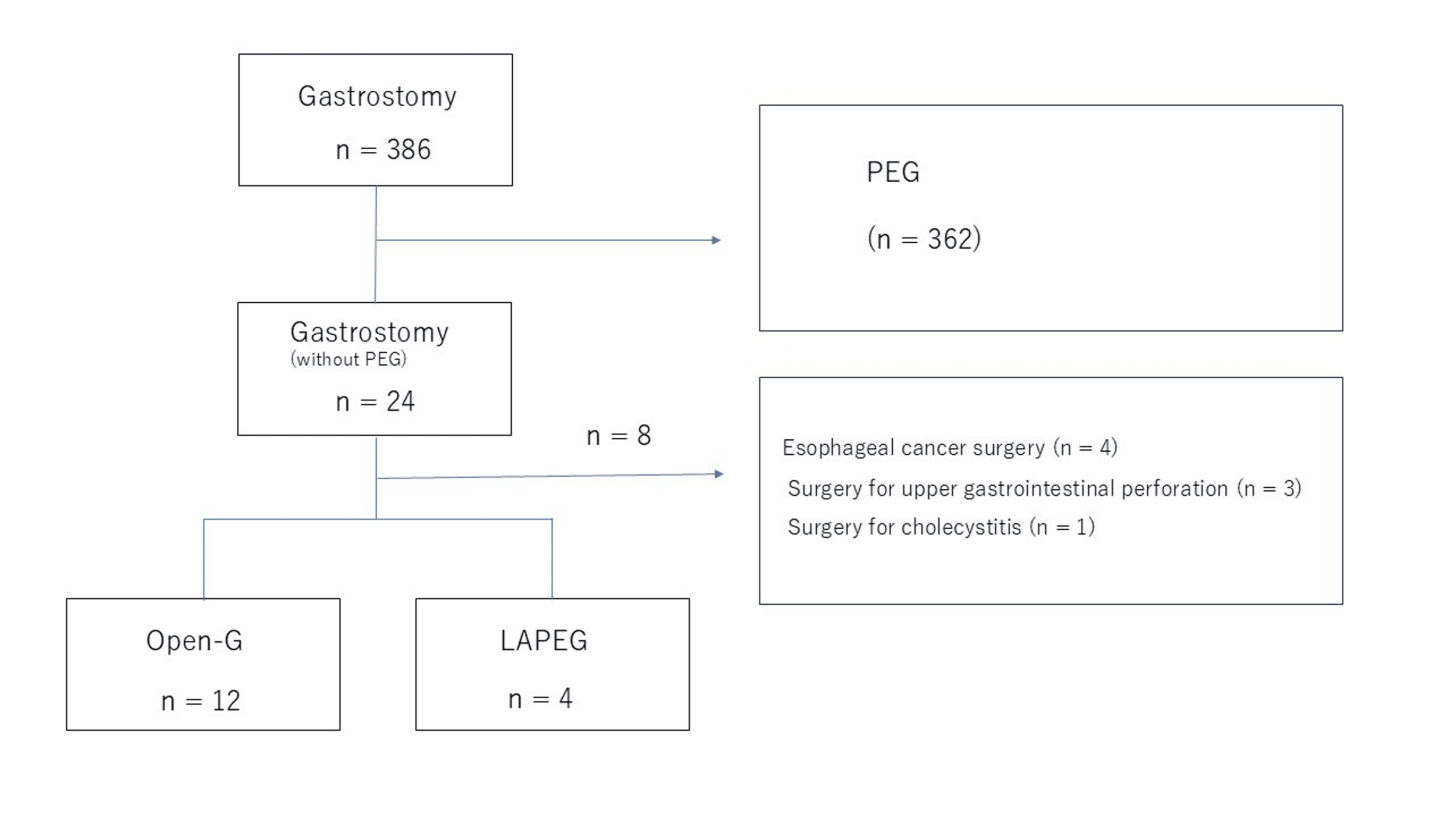
Study flowchart. Illustration of patient selection criteria and exclusion process for the study. The final analysis included patients who underwent gastrostomy and excluded those who required gastrostomy as part of another procedure. LAPEG, laparoscopically assisted percutaneous endoscopic gastrostomy; PEG, percutaneous endoscopic gastrostomy

A retrospective chart review was conducted for patients undergoing gastrostomy (tube placement) at Aizawa Hospital between January 2016 and February 2025. As shown in Figure 1, among 378 gastrostomy procedures, 16 were classified as *difficult percutaneous endoscopic gastrostomy*, defined by failure to confirm the transillumination or finger sign on endoscopy or by imaging evidence that an organ (e.g., transverse colon) overlapped the stomach. Patients requiring a gastrostomy as part of another concurrent procedure (e.g., combined surgeries) were excluded.

We examined patient demographics, baseline clinical status, and American Society of Anesthesiologists (ASA) classifications. Outcome measures included operative time, blood loss, and perioperative complications. The choice between Open-G and LAPEG depended on surgeons’ discretion, especially in earlier years when laparoscopic approaches were not fully standardized.

Statistical analyses were performed using t-tests with significance set at *P* < 0.05. All statistical analyses were performed using EZR, a graphical user interface for R (The R Foundation for Statistical Computing, Vienna, Austria).

Surgical procedures

Open-G

Under general anesthesia, a midline upper abdominal incision (typically 8-16 cm) was made. The stomach was visualized directly, and a 20-French balloon-type Foley catheter was introduced through the left lateral abdominal wall and fixed using the Stamm method. The gastric wall was sutured to the abdominal wall to ensure stability. About one month later, the balloon catheter was typically exchanged for a commercial button-type gastrostomy tube using a guidewire.

LAPEG

**Figure 2:**
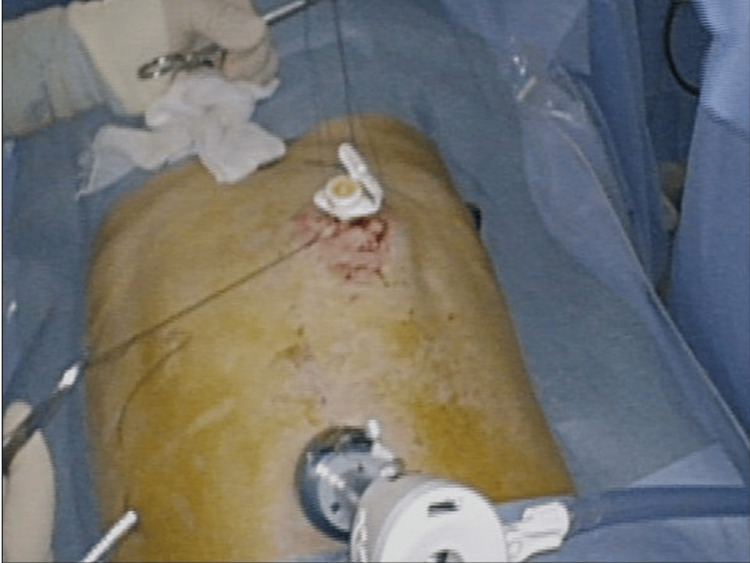
External view of the LAPEG procedure. External appearance during laparoscopically assisted percutaneous endoscopic gastrostomy (LAPEG) procedure, showing laparoscopic port placement.

LAPEG was performed under general anesthesia in the supine position. As shown in Figure 2, surgeons inserted two or three laparoscopic ports near the umbilicus.

**Figure 3:**
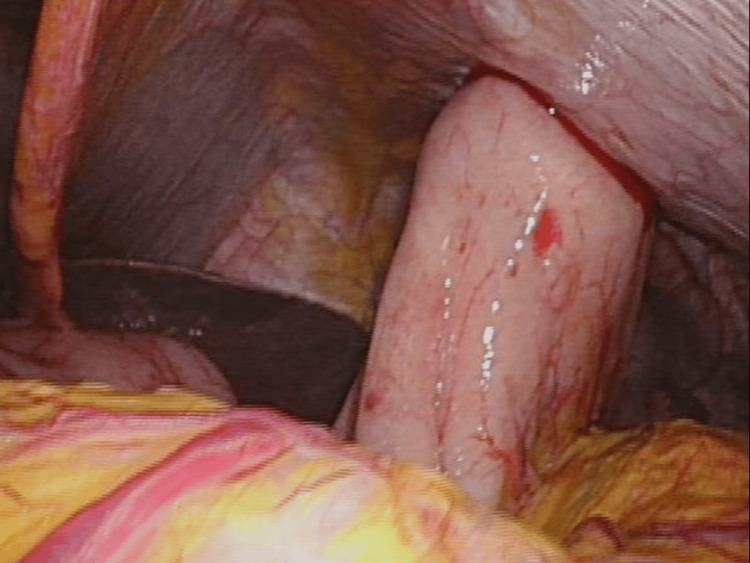
Internal view of the LAPEG procedure. Intraabdominal appearance of laparoscopically assisted percutaneous endoscopic gastrostomy (LAPEG) procedure. The gastrostomy button is inserted using the push technique under endoscopic guidance.

Adhesiolysis was carried out as needed. An endoscope was passed into the stomach, and the ideal gastrostomy site was identified under combined laparoscopic and endoscopic guidance. Figure 3 shows the intraabdominal appearance of the LAPEG procedure.

The *push* technique was used to place a button-type gastrostomy tube (EndoVive Bumper Button) during the initial procedure. Laparoscopic visualization minimized the risk of inadvertent injury to other organs.

## Results

Patient demographics

As shown in Table 1, 16 patients (four LAPEG and 12 Open-G) met the inclusion criteria.

**Table 1:** Patient demographics. Comparison of baseline characteristics between the LAPEG and Open-G groups. Variables include age, sex, underlying conditions, and reasons for PEG failure. No significant differences were observed between the two groups. LAPEG, laparoscopically assisted percutaneous endoscopic gastrostomy; Open-G, open gastrostomy

	Open-G (*n *= 12)	LAPEG (*n* = 4)
Sex (M/F)	8/4	4/0
Age, mean (range)	71.5 (61–86)	79 (74–85)
ASA classification (2/3)	7/5	2/2
Primary underlying disease		
Neurodegenerative disease	6	4
Cerebrovascular disease	3	
Head and neck cancer	2	
Hiatal hernia	1	
Reason for PEG difficulty		
Negative finger sign	5	2
Transverse colon overlying the stomach	3	2
Esophageal stricture after RT	1	
Esophageal perforation	1	
Sever hiatal hernia	1	
Tracheoesophageal fistula	1	

In the LAPEG group, all were male with a mean age of 79 years (range: 74-85 years). The most frequent underlying diagnoses were neurodegenerative diseases, such as Parkinson’s disease or progressive supranuclear palsy. The Open-G group comprised 12 patients (eight males, four females), with a mean age of 71 years (range: 61-86 years). Baseline characteristics, including ASA classification, showed no significant intergroup differences (Table 1).

Operative time and perioperative complications

Table 2 shows the operative time and perioperative complications of each group.

**Table 2:** Operative time and perioperative complications. Comparison of operative time, estimated blood loss, and postoperative complications between the LAPEG and Open-G groups. While the operative time and blood loss were comparable, complications were observed only in the Open-G group. LAPEG, laparoscopically assisted percutaneous endoscopic gastrostomy; Open-G, open gastrostomy

	Open-G (*n* = 12)	LAPEG (*n* = 4)	*P*-value
Operative time (minutes), median (range)	55.5 (43-75)	49 (42-94); 5 (2-10)	0.977
Blood loss (g), median (range)	5 (2-10)	3.5 (2-5)	0.121
Postoperative complications	3 (25%)	0 (%)	0.529
Bowel pneumatosis/paralytic ileus	2	-	
Aspiration pneumonia	1	-	

Median operative time was 49 minutes (LAPEG) versus 55.5 minutes (open), with no significant difference (*P* = 0.977). Blood loss was modest in both groups (3.5 g vs. 5 g) and did not differ significantly (*P* = 0.121). No perioperative complications occurred in the LAPEG group. In the open group, three complications were noted: two cases of bowel pneumatosis and one case of aspiration pneumonia. The frequency of these complications did not differ significantly between the groups.

## Discussion

Open-G was first reported by Stamm in 1894 as a method for long-term enteral feeding and gastric decompression. Subsequently, PEG was introduced by Gauderer et al. [1] in 1980 and rapidly gained widespread acceptance due to its minimally invasive nature, technical simplicity, and high success rate (exceeding 95%) [3-5].

However, many of the patients who require PEG have severe underlying diseases and limited physiological reserves, which can lead to a substantial risk of complications [6-7]. In particular, serious complications such as inadvertent puncture of the colon or small intestine and liver injury have been reported [8-9]. These events often occur at the time of gastrostomy placement but may initially go unnoticed.

To avoid injury to other organs, it is crucial to confirm transillumination by endoscopy (illumination sign) and the indentation caused by finger pressure on the abdominal wall (finger sign). In cases where these signs cannot be confirmed, the risk of accidental puncture of other organs increases, making safe PEG placement difficult. At our institution, confirming transillumination of the upper gastrointestinal endoscope through the abdominal wall, along with visible indentation by finger pressure under endoscopy, is a prerequisite for PEG. Patients not meeting these criteria, such as those with stenosis or perforation of the upper gastrointestinal tract, or CT findings indicating that the liver or transverse colon lies anterior to the stomach, are categorized as having *difficult PEG* and undergo surgical gastrostomy in the Department of Gastrointestinal Surgery.

We have traditionally performed Open-G for PEG-difficult patients. However, the need for a large incision and the cumbersome guidewire manipulation required when replacing a balloon catheter with a commercial kit have been noted as drawbacks.

LAPEG, first described by Raaf et al. [10], eliminates the risk of blind injury to abdominal organs and enables optimal selection of the gastrostomy site on both the stomach and anterior abdominal wall. By providing direct visualization under laparoscopy, the procedure allows for the stomach to be pulled into a normal position using minimal incisions, thereby avoiding organs that overlap the stomach. In addition, standard PEG kits can be used, simplifying post-procedure management [11-15].

In this study, we compared outcomes between LAPEG and Open-G in patients for whom PEG was deemed difficult. Although some reports suggest that LAPEG may reduce operative time and complications, the present analysis found no substantial difference between LAPEG and Open-G in terms of operative time or perioperative complication rates. However, the smaller incision in the LAPEG group may offer advantages in postoperative pain control and cosmetic outcomes.

In summary, when PEG is difficult, LAPEG offers certain practical advantages by allowing safe gastrostomy placement with the possibility of adhesiolysis and direct gastric re-positioning under laparoscopic guidance. Moreover, a smaller incision and the early use of button-type tubes may contribute to improved patient quality of life. However, given that our facility only recently standardized laparoscopic approaches, the choice between Open-G and LAPEG depended largely on the surgeon’s discretion. Consequently, selection bias cannot be ruled out, and larger-scale, prospective studies are needed to draw more definitive conclusions.

Limitations

This study was conducted at a single center with a small sample size, especially in the LAPEG group, which limits the statistical power. The decision-making process regarding the choice of Open-G or LAPEG was not governed by a strict protocol and may have been influenced by the lack of a standardized laparoscopic approach in our hospital until 2023. Future multicenter or prospective studies incorporating larger patient populations and more detailed operative data will be essential for a thorough evaluation.

## Conclusions

In this study comparing LAPEG and Open-G for patients with difficult PEG, there were no significant differences in operative time or perioperative complications. However, our sample size was small and the study is exploratory, limiting the strength of any firm conclusions. Given the potential advantages of smaller incisions, it remains a promising alternative for patients in whom standard PEG is not feasible. Further large-scale, prospective studies are warranted to clarify the clinical impacts of LAPEG and to guide optimal patient selection.
